# Fatigue Intervention by Nurses Evaluation – The FINE Trial. A randomised controlled trial of nurse led self-help treatment for patients in primary care with chronic fatigue syndrome: study protocol. [ISRCTN74156610]

**DOI:** 10.1186/1741-7015-4-9

**Published:** 2006-04-07

**Authors:** AJ Wearden, L Riste, C Dowrick, C Chew-Graham, RP Bentall, RK Morriss, S Peters, G Dunn, G Richardson, K Lovell, P Powell

**Affiliations:** 1 School of Psychological Sciences, University of Manchester, Manchester, M13 9PL, UK; 2 School of Population, Community and Behavioural Sciences, University of Liverpool, Liverpool, L69 3GB, UK; 3 Division of Primary Care, Rusholme Academic Unit, University of Manchester, Manchester, M14 5NP, UK; 4 Department of Psychiatry, Queens Medical Centre, University of Nottingham, Nottingham, NG7 2UH, UK; 5 Division of Psychiatry, University of Liverpool, Liverpool, L69 3GA, UK; 6 Division of Epidemiology and Health Sciences, University of Manchester, Manchester, M13 9PT, UK; 7 Centre for Health Economics, University of York, York, YO10 5DD, UK; 8 School of Nursing, Midwifery and Social Work, University of Manchester, Manchester, M13 9PL, UK; 9 Infectious Diseases Unit, Royal Liverpool University Hospital, Liverpool, L7 8XP UK

## Abstract

**Background:**

Chronic fatigue syndrome, also known as ME (CFS/ME), is a condition characterised primarily by severe, disabling fatigue, of unknown origin, which has a poor prognosis and serious personal and economic consequences. Evidence for the effectiveness of any treatment for CFS/ME in primary care, where most patients are seen, is sparse. Recently, a brief, pragmatic treatment for CFS/ME, based on a physiological dysregulation model of the condition, was shown to be successful in improving fatigue and physical functioning in patients in secondary care. The treatment involves providing patients with a readily understandable explanation of their symptoms, from which flows the rationale for a graded rehabilitative plan, developed collaboratively with the therapist. The present trial will test the effectiveness and cost-effectiveness of pragmatic rehabilitation when delivered by specially trained general nurses in primary care. We selected a client-centred counselling intervention, called supportive listening, as a comparison treatment. Counselling has been shown to be as effective as cognitive behaviour therapy for treating fatigue in primary care, is more readily available, and controls for supportive therapist contact time. Our control condition is treatment as usual by the general practitioner (GP).

**Methods and design:**

This study protocol describes the design of an ongoing, single-blind, pragmatic randomized controlled trial of a brief (18 week) self-help treatment, pragmatic rehabilitation, delivered by specially trained nurse-therapists in patients' homes, compared with nurse-therapist delivered supportive listening and treatment as usual by the GP. An economic evaluation, taking a societal viewpoint, is being carried out alongside the clinical trial. Three adult general nurses were trained over a six month period to deliver the two interventions. Patients aged over 18 and fulfilling the Oxford criteria for CFS are assessed at baseline, after the intervention, and again one year later. Primary outcomes are self-reported physical functioning and fatigue at one year, and will be analysed on an intention-to-treat basis. A qualitative study will examine the interventions' mechanisms of change, and also GPs' drivers and barriers towards referral.

## Background

Chronic fatigue syndrome, also known as ME (CFS/ME), is a symptomatically-defined condition in which the main complaint is medically unexplained fatigue, lasting at least six months, and of sufficient severity to impair the patient's functioning substantially [1,2]. The estimated prevalence in UK primary care ranges from 0.6–2.6%, depending on the criteria applied [3], while an Independent Working Party reporting to the UK Chief Medical Officer in 2002 suggested a UK population prevalence of 0.2–0.4% [4]. CFS/ME patients suffer more functional impairment than patients with Type 2 diabetes, acute myocardial infarction and multiple sclerosis [5]; they are also likely to stop working and to be heavy users of health care [6]. When conservatively treated, the prognosis for CFS/ME is poor [7,8]. The cost to society of failing to treat CFS/ME is considerable; in one primary care study, women with fatigue were over five times more likely to have no paid work than women in a matched comparison group [9].

A 2001 systematic review of all treatments for CFS/ME concluded that cognitive behaviour therapy (CBT) and graded exercise therapy (GET) were the most promising treatments for CFS/ME, but that owing to the small number of studies available for review, the generalisability of these results could not be assured [10]. The authors recommended that further studies be carried out using standardised outcome measures. A Cochrane Collaboration systematic review of 13 RCTs of CBT for CFS [11] reported that only 3 trials met inclusion criteria for the review. Of these three trials, two showed that hospital-based CBT was an effective treatment [12,13], a conclusion supported by a subsequent hospital-based trial of CBT [14]. Unfortunately, however, cognitive behaviour therapy is not a practical option in many parts of the UK because there are few therapists with the training and experience to carry it out [15]. Graded exercise therapy has also been shown to be effective in randomised controlled trials with selected hospital patients [16], and in our own previous study with a more general sample of hospital patients [17]. It is simpler than CBT to teach, but may not always be acceptable to patients [18]. Since the research described in this protocol was funded, two further trials of GET have been published, in secondary care or university settings, one of which showed improvements in global clinical outcome and fatigue [19], the other of which did not [20].

It has recently been suggested that more research is needed before we can be confident that effective secondary care treatments for CFS/ME can be extrapolated to primary care [21]. A randomised trial carried out in primary care compared CBT and counselling for chronic fatigue (28% of patients fulfilled criteria for CFS). Although the two treatments were found to be equally effective [22], the CBT was more expensive than counselling [23], and no treatment as usual comparison was included. Subsequently, in another equivalence trial, CBT and GET were compared in a primary care sample of chronically fatigued patients, 29% of whom met criteria for CFS. Again, the two treatments were found to be equally effective, but once again, they were not compared with a treatment as usual control group [18]. A self-help manual was shown to be more effective than no treatment for a community sample of patients with chronic fatigue, but only 18% of the sample fulfilled diagnostic criteria for CFS [24]. Most recently, and since the present trial was funded, a Dutch primary care trial has been reported in which general practitioners received brief training to deliver CBT to fatigued employees on sick leave, 44% of whom probably met research diagnostic criteria for CFS [25]. The CBT intervention was no more effective than GPs' usual care in reducing fatigue or returning patients to work.

To our knowledge, therefore, no adequately controlled trial of treatment for chronic fatigue syndrome in primary care has been published to date. Those treatments that have been shown to be effective in secondary care are not widely available. If a brief, readily communicable and easily implementable treatment could be shown to be clinically effective and cost effective in primary care, this would benefit the large proportion of patients who currently receive no effective treatment, including patients who might have difficulty accessing secondary care services.

We previously reported on a large-scale hospital-based trial in Liverpool, with out-patients suffering from CFS/ME, of a treatment termed "pragmatic rehabilitation" [26]. The treatment is based on a model proposing that CFS/ME is best understood as a consequence of physiological dysregulation associated with inactivity and disturbance of sleep and circadian rhythms [27]. We have argued that these conditions may be provoked by a number of biological and psychological factors but are often maintained by illness beliefs that lead to exercise-avoidance. The essential feature of the treatment is the provision of a detailed explanation for patients' symptoms, couched in terms of the physiological dysregulation model, from which flows the rationale for a graded return to activity. The explanations for various symptoms of CFS/ME highlight the interaction between psychological and biological factors. They are given to the patient both verbally during an initial discussion with the therapist and in the form of a fully-referenced manual, which has been developed in close collaboration with patients who have successfully completed the treatment. Having taken control of their symptoms through a programme of graded activity, normalisation of sleep patterns, and simple anxiety and stress-reducing procedures, patients are better able to consider the role of psychological and social factors in their condition.

In the Liverpool trial, three treatment groups received the manual and varying amounts of contact with a therapist over a three-month period; either nine face-to-face sessions (maximum treatment), two face-to-face sessions and seven telephone calls, or two face-to-face sessions only (minimum treatment). Results showed that all of these treatments were very effective when compared with medical assessment and advice: 57% of patients in the treatment groups no longer fulfilled case criteria for CFS 12 months after starting treatment, as compared with 6% of patients in the control condition [26]. Analysis of 2-year follow-up data has now taken place. This shows that, while all three treatment groups maintained substantial and clinically significant gains, there was a non-significant trend for those in the minimum treatment group to show deterioration in physical functioning and fatigue scale scores, suggesting that at least the medium amount of treatment should be given [28].

Our results [26] indicate that pragmatic rehabilitation is an effective treatment for hospital-based patients. The treatment trial protocol reported here was designed to determine whether pragmatic rehabilitation is effective in primary care settings, when delivered by non-specialist nursing staff who have received brief training. We decided to test pragmatic rehabilitation against two treatments, a patient-centred counselling treatment called supportive listening, and treatment as usual by the GP. As noted above, counselling has been shown to be as effective as CBT for chronic fatigue in primary care [22], but has not been tested for chronic fatigue syndrome. It is a treatment from which patients can be expected to benefit and will also control for supportive therapist contact time.

### Aims of the study

This study was designed to address four principal research questions:

(1) Is pragmatic rehabilitation, delivered at home by nurses to CFS/ME patients recruited from primary care, a clinically effective intervention in terms of reduced disability and fatigue when compared with treatment as usual delivered through the primary care team?

(2) Is supportive listening, delivered at home by nurses to CFS/ME patients recruited from primary care, a clinically effective intervention in terms of reduced disability and fatigue when compared with treatment as usual delivered through the primary care team?

(3) Which of the three treatments, pragmatic rehabilitation, supportive listening and treatment as usual, is the most cost-effective?

The study will also address three further issues: the relative efficacy and cost-effectiveness of pragmatic rehabilitation and supportive listening; the mechanism of action of these treatments; and barriers to the implementation of these treatments in UK primary care settings.

## Methods and design

### Design

This is a single-blind pragmatic randomised controlled trial of a brief (18-week) self-help treatment, called pragmatic rehabilitation, for CFS/ME patients recruited from primary care, delivered by three specially trained nurse-therapists in the patients' homes, and compared with both nurse-therapist delivered supportive listening, a comparison treatment that also controls for therapist contact time, and treatment as usual by the General Practitioner.

### Setting

The study is currently taking place across the North West of England. Patients are referred to the trial by their general practitioners and assessed in their own homes. If randomised to pragmatic rehabilitation or supportive listening, patients are treated at home.

### Ethical approval

This study has been reviewed and approved by the Eastern MREC (reference 03/5/62)

### Training the nurse therapists to deliver the interventions

The three nurse-therapists delivering the pragmatic rehabilitation and supportive listening treatments were employed specifically to work on this trial. We decided to recruit three nurses (adult registered general nurses, RGN) with an interest in psychosocial aspects of health care (e.g. district nurses, practice nurses), who were used to working in the community and primary care, and who could manage medical co-morbidity, in order to develop a treatment that could be made widely available. Primary care general nurse practitioners have been successfully trained to implement interventions based on cognitive behaviour therapy for patients who were high utilisers of medical care and had medically unexplained symptoms [29] and irritable bowel syndrome [30]. We had experience of training practice nurses to assess and manage anxiety disorders [31], and of training mental health professionals, including nurses, to deliver individual problem-solving treatment and a group educational programme to primary care patients with depression [32]. In addition, we had experience of training a non-specialist nurse to deliver pragmatic rehabilitation in the Liverpool hospital-based service.

Training of the nurse therapists to deliver both interventions took place over a six-month period. Training in each intervention took place over a period of four months. Training in the supportive listening intervention started first. After two months, training in the pragmatic rehabilitation intervention started, so that there was a period of overlap when both interventions were being taught and practised together.

Training in the supportive listening treatment was provided by an experienced counsellor with expertise in the client-centred counselling approach, and was supported by a training manual specially written for this study. Sixteen half-days of training were provided, in which the nurse-therapists were introduced to the techniques and concepts of client-centred counselling, and developed and practised new skills using exercises, discussions and role-plays.

Sixteen half days of training in the techniques of pragmatic rehabilitation were delivered over a twelve week period by members of the trial team, working with the extensive training materials developed by Dr. Pauline Powell. A particular focus of the pragmatic rehabilitation training was on teaching the nurses to work with the patient manual and on equipping the nurses with the necessary skills to enable patients to use the manual effectively. Audio-taped role-play and other practical exercises were used. During the training period, the nurse-therapists also "shadowed" Pauline Powell and her co-workers in the hospital setting in order to gain additional experience.

The nurse therapists were provided with diaries to record their achievements during the training period. Before the nurses started treating patients, they practised the skills that they had learned in training by delivering the scheduled treatments to four volunteer patients each. These sessions were audiotaped (with the patients' permission) and the tapes, together with material generated by the patients and the nurses, were assessed to evaluate nurses' practice in accordance with pre-defined criteria relating to knowledge, skills and attitude [31].

The nurse therapists are receiving regular supervision while they are treating patients. For the pragmatic rehabilitation arm, supervision is provided by two members of the trial team, while supervision for the supportive listening arm is being provided by a counsellor experienced in supervision. In both arms, there is a mixture of group and individual supervision sessions, and supervision takes place at approximately two-weekly intervals. Booster training sessions are being provided in both treatment arms at approximately four-monthly intervals.

### The participants – inclusion and exclusion criteria

Patients aged 18 and over who fulfil the Oxford criteria for CFS [1] are eligible for inclusion in the trial, with no upper age limit. These criteria require that patients have a principal complaint of severe, unexplained fatigue, of new onset, persisting or relapsing over a period of at least six months, which results in a substantial impairment to previous levels of activity and affects both mental and physical functioning. We have chosen the Oxford criteria for inclusion as they are simpler for GPs to operate than the CDC criteria [2], but at baseline assessment we also ascertain whether patients meet the slightly more restrictive CDC diagnostic criteria. The CDC criteria, in addition to the above, require that patients have at least four from a list of eight other symptoms, concurrent with the fatigue [2]. We also record whether patients fulfil London ME criteria at baseline [33]. We decided not to use the recently published Canadian criteria [34], as these have not yet been validated for research purposes. Both the CDC and the Oxford criteria require that possible medical and psychiatric causes for fatigue are excluded by the taking of a thorough history, physical examination and a battery of tests. Participating GPs are provided with a brief protocol to enable them to carry out these assessments. As GPs may experience difficulty in determining patients' levels of activity, we require that patients are sufficiently functionally impaired to score 70% or less on the SF-36 physical functioning scale [35]. Additionally, patients are required to score 4 or more on the 11-item Chalder fatigue scale [36]. In accordance with the Oxford and CDC criteria [1,2], patients whose fatigue is explained by any active medical condition are excluded, as are patients with schizophrenia, bipolar disorder, dementia, eating disorders or substance abuse. Patients with active suicidal ideation (i.e. within the last month) or with antisocial, borderline or paranoid personality disorders are excluded because our nurse therapists are not trained to treat these conditions. The following patients are also excluded: patients who cannot read or write English sufficiently well to participate in treatment or complete the assessment measures; patients who are incapable of giving informed consent; patients who are currently undertaking systematic psychological therapies for CFS/ME or who have received pragmatic rehabilitation therapy within the past year. Patients with mild to moderate depression and/or anxiety disorders are not excluded. Patients with comorbid conditions (for example, orthopaedic problems) that might be exacerbated by the activity programme are not excluded, but the advice given to them is tailored accordingly.

### The interventions

There are three treatment arms: pragmatic rehabilitation, supportive listening, and treatment as usual by the general practitioner. Three nurse-therapists deliver the pragmatic rehabilitation and supportive listening treatments in patients' homes and via telephone calls. All three nurse-therapists deliver both treatments. Each patient in the pragmatic rehabilitation and supportive listening arms is treated by only one nurse therapist. The schedule of therapist contact is identical in the two treatment arms. The treatment period is eighteen weeks.

Pragmatic rehabilitation and supportive listening treatments each consist of five face-to-face sessions delivered in the patient's home (on weeks 1, 2, 4, 10 and 19), interspersed with five 30-minute telephone calls to the patient (on weeks 3, 6, 8, 12 and 15), in accordance with a standard operating protocol. The first face-to-face session lasts approximately 90 minutes, while the other four face to face sessions last approximately 60 minutes. The telephone calls last a maximum of 30 minutes. A record is kept of the dates on which treatment sessions are delivered, and of the length of each session.

We needed a longer first face-to-face session to allow time for the delivery of the rationale for pragmatic rehabilitation and supportive listening. While two face-to-face sessions with the therapist were effective in the Liverpool secondary care trial [26], patients in that trial first met with the therapist after an extended consultation with the consultant physician, which may have had therapeutic effects. We therefore felt that we needed at least one extra face-to-face session with the therapist, and decided to have the fourth home visit in order to be able to treat the more complex or chronic presentations. The fifth home visit is a discharge visit and focuses on identifying further treatment needs and planning for the future. The nurse therapists keep diaries of their activities during treatment, and these are used during supervision. We piloted the timing of the sessions and telephone calls with volunteer training patients before trial treatment started and found that these timings were acceptable to both the patients and the nurse therapists.

#### The content of pragmatic rehabilitation treatment

The first intervention session is taken up with providing patients with a detailed explanation of their symptoms in terms of such physiological explanations as circadian rhythm desynchronisation, disrupted sleep patterns, neuro-endocrinological disturbances, and cardiovascular and muscular deconditioning. The somatic manifestations of anxiety are also explained to the patient. The patient is given the manual at this session. Our experience is that this initial session, which provides patients with convincing explanations for their symptoms and also indicates how they can be targeted in treatment, is crucial to the success of the intervention; in the Liverpool trial the majority of patients rated this component as very helpful on a follow-up questionnaire [37]. Patients are then encouraged to embark upon a series of treatment components, including graded exercise (starting at a very low level and increased very gradually), a return to more regular sleep patterns, and relaxation exercises. The initial level of activity is determined collaboratively with the patient, taking into account the patients' current activity levels, and always starts at a level less taxing than the patient can currently manage. During meetings and telephone calls with the therapist, the rationale for the treatment is reinforced, and the patient encouraged to adhere to treatment using motivational interviewing techniques [38]. Patients are asked to keep a diary of their progress on the exercise programme, together with a note of their daily activities, rest and sleep, and any problems encountered and how they were dealt with. These diaries, which remain the property of the patient, are then reviewed at each contact with the nurse.

#### The content of supportive listening treatment

We recognise that patients with CFS/ME often feel misunderstood or disbelieved, so an important part of any non-specific treatment effect might be attributable to the patient feeling that his or her symptoms are being taken seriously, and that his or her status as an ill person is accepted. The aim of the supportive listening treatment arm is to provide emotional support and validation for the patient through the development of a collaborative relationship in which the patient is held in unconditional positive regard, and encouraged to talk about his or her experience of CFS/ME and problems which he or she has in dealing with it. The content of patient-therapist discussions is largely determined by the patient. In order to provide parity with the pragmatic rehabilitation intervention, patients in the supportive listening arm are provided with a short manual that explains the principles of client-centred counselling, provides suggestions as to how to get the most out the treatment, and includes diary pages for the patient to record his or her own thoughts and feelings.

#### The content of treatment as usual by the General Practitioner

GPs usually try to talk to patients such as these about their symptoms in an unstructured way, investigate, provide reassurance, prescribe symptomatic relief, prescribe hypnotics, sedatives and anti-depressants, and refer. For this trial, GPs are advised on how to assess CFS/ME patients, and asked not to refer to specialist services for systematic psychological therapies for CFS/ME during the eighteen week treatment period, but are otherwise invited to manage their allocated cases as usual in the way that they see fit. Treatment received by patients from their GPs will be determined at the end of the trial by examination of GP records, and patients will also be asked about the care they received at the 20 and 70 week assessments.

#### Treatment fidelity

All three therapists deliver both the pragmatic rehabilitation and the supportive listening treatments. We chose this approach rather than having different therapists delivering the different therapies in order to reduce the confounding of therapist effects. We have a good track record in trials of CFS/ME [17] and other similar conditions [39] of achieving fidelity when the same therapist delivers more than one treatment, and we recognise the importance of maintaining treatment fidelity in this trial. With patients' permission, all nurse therapist visits and telephone calls are audio-taped, using digital recorders. We have developed a schedule for treatment fidelity ratings and have recruited three independent raters who are rating a random sample (5%) of the therapy tapes. The tapes are selected so that: (1) approximately equal numbers come from each therapist, (2) approximately equal numbers of PR and SL are represented and (3) all treatment sessions are represented, but are otherwise selected at random. Tapes are rated blind to treatment classification. Each tape is classified as one or other treatment, and receives an overall quality of therapy rating. Additionally, more detailed ratings of therapeutic alliance, generic therapy quality and treatment-specific therapy quality are produced, which will be used in subsidiary analyses.

#### Treatment adherence

Experience from the Liverpool study suggests that patients who accepted the rationale for treatment were willing to adhere to it. In the Liverpool study, treatment was completed by 127/148 patients; i.e. a drop-out rate of only 14%. On the other hand, in a trial of cognitive behaviour therapy versus counselling for fatigue in primary care [22], 44/193 (35%) eligible patients failed to complete treatment, suggesting that the likely degree of adherence in primary care may be lower than that seen in hospital care.

#### Anticipated loss to follow up

In the Liverpool study, 148 patients were randomised to treatments; each was scheduled to receive four assessments by postal questionnaire (baseline, 3 months, 6 months, one year), making a total of 592 assessments in all. At the end of the trial, 541 of these assessments had been completed (including those completed on drop-outs), giving a loss to follow up of only 9%. Our patients will be required to complete a larger battery of assessments than those in the Liverpool study, which might reduce participation rates. On the other hand, we anticipate that the fact that interventions and assessment will take place in the patients' homes in this trial will reduce both failure to adhere to treatment and failure to complete assessments. To ensure that we have sufficient numbers to test our hypotheses, we have estimated a rate of 23% combined non-compliance and loss to follow-up (this being half-way between the 35% drop out rate reported in a previous primary care trial [22], and the 9% loss to follow up in our previous trial). To reduce loss to follow-up, we plan to ask patients to nominate a relative or friend who could be contacted to help us to trace them should they move away.

Some CFS/ME patient support groups have suggested that activity may be harmful to some CFS/ME patients [4], but no adverse outcomes have been reported from previous well-conducted treatment trials that have included adequately graded activity as a component of treatment [4]. The activity programme forming part of pragmatic rehabilitation is determined collaboratively by the patient and therapist, and always starts at a level less taxing than the patient can currently manage. Similarly, the rate at which activity is increased and the type of activity undertaken are determined by the patient in collaboration with the therapist. If there are any adverse reactions, these will be notified to the independent committees overseeing the trial (the Data Monitoring and Ethics and Trial Steering Committees) and to MREC, in accordance with agreed procedures.

### Recruitment of patients

**Figure 1:**
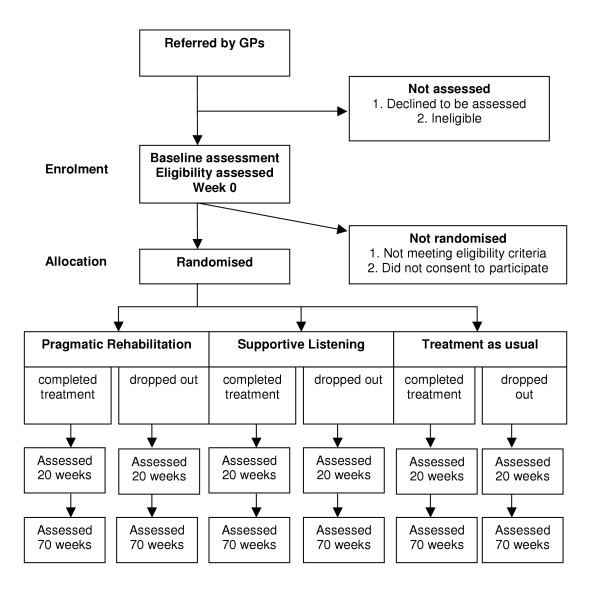
CONSORT diagram.

After obtaining formal approval from the relevant Primary Care Trusts, information about the trial is sent to GP practices in the North West of England. GPs are asked to identify patients whom they think may be suitable for the trial from their patient lists and from new consultations, and then to assess each patient in accordance with a protocol/checklist provided in their information pack. The checklist is based on the Oxford criteria [1] and includes a list of advised exclusionary tests. If, in the GP's opinion, the patient fulfils the trial criteria, and the patient agrees, a referral is sent to the FINE Trial office. A copy of the Patient Information Sheet and consent form is sent from the FINE Trial office to the patient, and followed up one week later by a telephone call from one of the research team (research assistant or trial manager) during which the patient can ask questions and decide whether he or she wishes to be assessed. At the baseline assessment, which takes place in the patient's home, the trial is explained again, the importance of maintaining researcher blindness is emphasised to the patient, and written informed consent is obtained. The baseline assessment is then carried out. (See Figure 1).

### The baseline assessment

The baseline assessment serves two purposes – firstly to ensure that patients recruited to the trial meet study criteria, and secondly to obtain baseline measures on all our outcomes. The schedule for the baseline assessment and subsequent assessments is shown in Table 1. If there is any doubt as to whether the patient fulfils criteria for entry to the study, the research assistant consults with medically qualified members of the team and/or the referring GP, and if necessary the patient can be referred back to the GP for further information or tests. Once we are satisfied that the patient fulfils study criteria, the trial manager notifies the randomisation service.

### Randomisation

Randomisation of individual patients as they are recruited to the trial is carried out by an independent randomisation unit based at Christie Hospital in Manchester, in consultation with the trial statistician, and in accordance with a randomisation protocol. The procedure uses randomised permuted blocks (with randomly-varying block sizes of 9, 12, 15 or 18), after stratification based on whether the patient is ambulatory or not (not ambulatory being defined as using a mobility aid on most days), and on whether the patient fulfils London ME criteria or not. There are therefore four strata in total. Where applicable, patients are assigned to therapists in a simple random fashion according to the therapists' availability. The trial manager is informed of randomisation, and group membership is conveyed to the designated nurse-therapist, if appropriate. The GP is informed of randomization and sent a copy of the patient consent form.

### Outcome measures

Patients are assessed at entry to the trial (week 0), within one week of the end of treatment (20 weeks), and then one year after finishing treatment (70 weeks). Assessments are carried out by trained researchers who are blind to the arm to which the patient has been randomised. Assessment at week 70 is required because short-term assessments of outcome in a chronic health condition such as CFS/ME can be misleading. Additionally, psychological treatments such as CBT become more effective over time [13] and we wish to be able to determine whether that is the case for our interventions. The primary outcome point is 70 weeks.

#### Primary outcome measures

The primary outcome measures are patient-rated to avoid observer bias. These are (1) score on the physical functioning scale of the SF-36 [35], (2) cost-effectiveness using the Euroqol [40] and (3) the score on the 11-item Fatigue Scale [36]. An improvement of 50% or more on the SF-36 physical functioning scale, or a score of 75% or more on that scale, will be considered a clinically significant improvement.

#### Secondary outcome measures

Secondary outcome measures are (1) time to take 20 steps, (or number of steps taken, if this is not achieved) and maximum heart rate reached on a step-test [41]; (ii) scores on the anxiety and depression scales of the HADS [42] to provide measures of depression and anxiety; (iii) scores a brief four-item sleep scale [43].

#### Predictors of response to treatment and measures of processes of change

In the Liverpool study, scores on the HADS predicted changes in the primary outcomes, physical functioning and fatigue. As lack of social support has been implicated in symptom persistence in chronic fatigue syndrome [44], we included a brief social support measure [45]. We administer a visual analogue measure of expectation of treatment after randomization but before treatment starts. Beliefs about CFS/ME, particularly physical illness attributions, predict response to treatment in primary care samples of patients with fatigue [46]. We administer brief questionnaire measures of beliefs about illness and the processes of supportive listening, designed especially for this study, but based on measures used in the Liverpool Hospital trial, at each assessment. These brief belief measures are also administered after the first home visit, to enable us to assess the impact of the first, rationale-giving session. We also administer questionnaire measures of symptom interpretation (Moss-Morris, R & Chalder T, personal communication) and therapeutic alliance [47]. Our qualitative study will provide a more detailed understanding of the processes of change.

### Qualitative study

Four phases of qualitative interviews are being carried out with sub-samples of patients and GPs. These will explore GP and patient attitudes to CFS/ME, its current management, and the trial interventions. In addition, we will determine the mechanisms of action of the interventions and identify barriers to their implementation. The composition of the samples will mirror that of the quantitative study, with representatives of all three treatment groups, study participators and study refusers, while the exact size of the sub-samples will be determined during the course of analysis using the constant comparative technique [48]. The first patient sub-sample will include patients who have consented to take part in the study. We will use maximum variance sampling to generate a range of respondents in terms of age, gender, sociodemographics and ethnicity, and the sample will include patients entering each of the three treatment groups. If possible, we will also interview patients who have refused to take part in the study, but consented to be interviewed. We anticipate that approximately 40 interviews will be required. These first phase qualitative interviews, carried out after the first quantitative assessment but before treatment commences, will explore patient views on illness causation, beliefs about CFS/ME, expectations of intervention and previous experience of treatment and doctor-patient relationships, and will provide a comparison for data obtained from the post-trial qualitative interviews. A second set of interviews will be carried out towards the end of the trial, with a different subset of patients, in order to investigate the processes related to successful engagement in and response to treatment. Sub-samples of patients from the three intervention groups with good and poor outcomes on final assessment will be selected (approximately 20 of each) and will be interviewed about their experiences of the intervention, the value they place on treatment, their relationship with the therapist, and whether their views on the causation of CFS/ME changed during the course of treatment. Patients' views on the effectiveness of the treatment and their plans for management of future health problems will be sought. Particular attention will be paid to equality issues in terms of access to and experience of treatment. Thirdly, qualitative interviews with a representative sample of GPs who have referred or not referred patients to the study (approximately 10 of each) will be performed to explore barriers and drivers towards referral. Finally, the three nurse therapists were interviewed at the end of training and prior to starting treatment, and will be interviewed again at the end of the trial to ascertain their experience of being trained to deliver these treatments, and of the treatment process itself.

### The economic evaluation

A full economic evaluation is being undertaken alongside the clinical trial. The economic evaluation takes a societal viewpoint, but will also disaggregate data to enable us to examine implications for specific sectors such as primary and secondary care within the NHS as well other government agencies (for example Social Services). Resource-use data detailing the use of primary and secondary care services, social and community services, private and voluntary services are collected at each assessment point. Unit cost estimates will then be applied to each resource, using figures from local providers supplemented by national data where appropriate. For each patient in the trial, a total cost will be estimated by calculating the product of resource use and the relevant unit cost. In addition, cost to patients (e.g. travel costs) will be estimated at 70 weeks. Cost data will be analysed using appropriate statistical techniques, such as bootstrapping, and will also be subjected to extensive sensitivity analysis. A cost-utility analysis will be performed using the EQ5D [40] as a measure of health related quality of life.

The power analysis for this trial has been based on the effects expected in the clinical measures. To address the issue of the adequacy of the sample size to perform the economic evaluation, methods of presenting data that allow for the uncertainty created by potentially under-powered economic evaluations, such as cost-effectiveness acceptability curves, will be employed. These techniques allow for the cost of the uncertainty to be estimated and therefore the value of future data collection can be assessed.

### Power analysis

We plan to recruit 120 patients for each treatment arm. Our power calculation was based on a comparison between pragmatic rehabilitation and treatment as usual. In the Liverpool hospital-based study [26], at 12 months after the start of treatment, on an intention to treat basis with previous scores assigned for dropouts, 57% of intervention group patients and 6% of control group patients were classified as "no longer cases" (i.e. scored 4 or less on the Fatigue Scale and 75% or more on the SF36 physical functioning scale). On the basis of previous studies, we would expect to see a bigger improvement in patients receiving treatment as usual in primary care [7,9]. We have also taken a more conservative estimate of the proportion of patients likely to improve in the pragmatic rehabilitation condition; we believe that a 20% difference in improvement rates between intervention and control patients would be of interest to the NHS. A two-group χ^2 ^test with a 0.05 two-sided significance level will have 80% power to detect the difference between an intervention group proportion of 50% improved and a treatment as usual group proportion of 30% improved, when the final sample size is 93 per group. Allowing for a rate of loss to follow-up of 23% in the treatment as usual group, the sample size required is 120 per group.

### Analysis plan

Analysis of both the quantitative and qualitative data collected during the course of the trial will take place in accordance with pre-determined analysis plans, which have been lodged with the Data Monitoring and Ethics and Trial Steering Committees.

#### Analysis of quantitative data

Baseline comparability of the three treatment groups will be described, but no significance testing will be conducted. Acceptance of and adherence to treatment will be described. Patients in the pragmatic rehabilitation and supportive listening arms will be deemed to have accepted treatment allocation if, after randomization, they embark on treatment, that is they make themselves available for the first treatment session. Patients in the treatment as usual arm will be deemed to have accepted treatment if, after randomization, they agree to return to their GP's care. To measure adherence to treatment, in the pragmatic rehabilitation and supportive listening arms, the number of home visit sessions and number of telephone calls that in the opinion of the therapist constituted an intervention session will be counted. No attempt will be made to assess adherence further than this. In the absence of any protocol violation (e.g. receiving a systematic psychological therapy for CFS/ME), patients in the treatment as usual arm will be deemed to have adhered to treatment. Missing outcome data will be described in relation to patient baseline characteristics and adherence to treatment.

Descriptive statistics for outcome measures will be presented. The main analyses will be carried out using all patients randomized using the principle of Intention-To-Treat (ITT). With three groups (Pragmatic Rehabilitation – PR, Supportive Listening – SL and Treatment as Usual – TAU) we will have two degrees of freedom to test for group differences. Following a global test to determine whether the three groups are different, pragmatic rehabilitation will be contrasted with supportive listening. If there is no evidence of any difference in outcome between pragmatic rehabilitation and supportive listening, they will be jointly contrasted with treatment as usual. If pragmatic rehabilitation and supportive listening differ in their outcomes, then they will be separately compared with treatment as usual. Effect sizes and confidence intervals will be reported for each group

Sensitivity of the treatment effect estimates to potential biases arising from missing outcome data will be evaluated by the use of inverse probability weighting [49,50]. This assumes that the missing data mechanism is ignorable – i.e. Missing at Random (MAR). We will also carry out a likelihood-based analysis of all available data using *Mplus *[51] again assuming that the missing data mechanism is ignorable.

A supplementary set of analyses will be undertaken to assess the effect of non-adherence to allocated treatment (again taking account of sensitivity to assumed missing data mechanisms) in order to estimate treatment effects in those patients who have complied with randomization (the estimation of the Complier Average Causal Effect – CACE – as opposed to the Average Causal Effect – ACE – as estimated using ITT methods). We have provided detailed examples of this approach elsewhere [52,53].

All analyses to estimate treatment effects will be variations on an analysis of covariance with the exact form depending on whether outcome is binary or quantitative. Covariates will include whether the patient is ambulatory or not, and whether the patient fulfils London ME criteria or not.

Supplementary, exploratory analyses will be carried out to determine predictors of response to treatment. These will include, but may not be restricted to, (1) dichotomous variables such as membership of self-help group, whether ambulatory or not, whether the patient fulfilled London ME criteria, presence or absence of psychiatric diagnosis (e.g. depression); and (2) continuous variables such as illness duration, score on HAD depression scale, score on social support scale, and baseline illness beliefs scores. Analysis will take place once, after data collection is complete. There are no proposed interim analyses.

### Trial management and oversight

The day to day management of the trial is carried out by Trial Manager, Dr Lisa Riste, in consultation with the Principal Investigator, Dr Alison Wearden, and other members of the trial team as necessary. A Trial Management Group consisting of the grant holders (including Professor Graham Dunn, the trial statistician), the trial manager and research assistants meets monthly. Additional meetings are held to discuss aspects of the pragmatic rehabilitation and supportive listening therapies, as necessary. Monthly meetings to monitor trial recruitment are held. The trial is advised by an independent Trial Steering Committee and overseen by an independent Data Monitoring Ethics Committee, both of which meet yearly and operate in accordance with Medical Research Council Guidelines.

## Discussion

Our trial will be the first randomised controlled trial of treatments for CFS/ME in primary care, and will add to the knowledge base in a number of ways. We have both a comparison treatment that controls for supportive therapist contact time and a treatment as usual control arm, which will help us to assess the efficacy of pragmatic rehabilitation. Our trial is conservatively powered, so we can be confident of detecting clinically significant effects. This is a pragmatic trial with relatively inclusive entry criteria, and we anticipate recruiting patients from across the spectrum of illness severity. Because we are treating patients in their homes, we are able to assess and treat patients who are unable to access secondary care services. These features will improve the generalisability of our findings.

We have taken a number of steps to protect our trial against sources of bias. While therapist effects are not a primary outcome measure in this trial, we have chosen to use three nurses in order to minimise, or at least observe, possible therapist effects. To counteract possible biases introduced by the place of recruitment, all three nurses treat patients recruited from across the recruitment area. Differential willingness of patients to be randomised to treatment as usual is minimised by constraining only systematic psychological therapies for CFS/ME in the treatment as usual arm of the trial. To reduce observer bias in assessment, all assessment data are collected by research assistants not involved in, and blind to, treatment; in addition, the research assistants are accommodated in different locations from the therapists. All unblindings are recorded systematically. All outcome measures except the step-test are patient-rated. Bias and random error in handling data will be minimised by validation checks by the trial manager on data entry by the research assistants.

If pragmatic rehabilitation is successful in producing a specific and lasting cost effective clinical benefit, we expect our findings to have a major impact on training and services in primary care. We will use our results to develop educational programmes for primary care health professionals. If our treatments prove effective and cost-effective in primary care, we envisage that they would be deliverable as specialist services provided or commissioned by primary care trusts. We have taken steps to ensure that our training of nurse therapists in the two treatments is well-described and replicable, so that training and service delivery can be implemented later.

## Competing interests

The author(s) declare that they have no competing interests.

## Authors' contributions

All authors contributed to the overall design of this study, and are involved in the ongoing management of the trial. AW is the principal investigator with overall responsibility for the FINE Trial. She conceived of the study, participated in its design and coordination, prepared the protocol, and participated in the training of nurse therapists. LR is the trial manager. She participated in staff training, is responsible for recruitment to the trial, staff management and coordination of the study. CD participated in the design and coordination of the study, the training of the nurse therapists, and the recruitment of general practices into the study. CCG is responsible for developing and overseeing the qualitative components of the trial, and assisting in the recruitment of general practices to the trial. RPB developed pragmatic rehabilitation treatment in secondary care, participated in the development of the supportive listening treatment, and participated in the training and ongoing supervision of nurse therapists. RKM participated in the training and ongoing supervision of nurse therapists, trained the researchers in psychiatric interviewing and operation of the inclusion and exclusion criteria, and contributed to the design of the qualitative study. SP is responsible for developing and overseeing the qualitative components of the trial, and assisting in the recruitment of patients to the trial. GD is the trial statistician. He advised on randomisation and all statistical aspects of the trial and developed the analysis plan. GR developed the health economic study and the health economics analysis plan. KL participated in the design of the therapy rating exercise and has advised on recruitment of patients into the trial. PP developed pragmatic rehabilitation treatment and the patient pragmatic rehabilitation manual in secondary care, participated in the training of the nurse therapists, and advised on the adaptation of pragmatic rehabilitation for the primary care setting. All authors read and approved the final manuscript.

## Pre-publication history

The pre-publication history for this paper can be accessed here:



## Figures and Tables

**Table 1:** Schedule of assessments and measures administered to patients

Measures	Week 0	Week 0	Week 1	Week 10	Weeks 20,70
I = interview SRQ = self-report questionnaire O = other	Baseline assessment pre-randomization	Post-randomization	Following first intervention visit	Following week 10 home visit	Post-treatment assessments

Demographics [I]	+	-	-	-	-
Medical history [I]	+	-	-	-	-
Oxford criteria [I][1]	+	-	-	-	+
CDC criteria [I][2]	+	-	-	-	+
London ME criteria [I][33]	+	-	-	-	+
SF-36 physical functioning scale [SRQ][35]	+	-	-	-	+
Chalder Fatigue scale [SRQ] [36]	+	-	-	-	+
Hospital Anxiety and Depression Scale [SRQ] [42]	+	-	-	-	+
Sleep scale [SRQ][43]	+	-	-	-	+
Euroqol [SRQ][40]	+	-	-	-	+
SCID screen for alcohol/substance abuse [I][54]	+	-	-	-	-
SCID screen for personality disorders [I][55]	+	-	-	-	-
SCID depression and anxiety diagnoses [I][54]	+	-	-	-	+
Brief belief measure [SRQ]	+	-	-	-	+
Brief SL process measure [SRQ]	+	-	-	-	+
Brief social support measure [SRQ][45]	+	-	-	-	+
Symptom interpretation questionnaire [SRQ]	+	-	-	-	+
Economic assessment (resource use)[I]	+	-	-	-	+
Step test [O][41]	+	-	-	-	+
Visual analogue scale: treatment expectation [SRQ]	-	+	-	-	-
CALPAS measure of therapeutic alliance [SRQ]	-	-	+	+	+
